# Cardiorenal Syndrome Type 5: *In Vitro* Cytotoxicity Effects on Renal Tubular Cells and Inflammatory Profile

**DOI:** 10.1155/2015/469461

**Published:** 2015-07-22

**Authors:** Alessandra Brocca, Grazia Maria Virzì, Chiara Pasqualin, Silvia Pastori, Stefano Marcante, Massimo de Cal, Claudio Ronco

**Affiliations:** ^1^Department of Nephrology, Dialysis and Transplantation, International Renal Research Institute of Vicenza (IRRIV), San Bortolo Hospital, Via Rodolfi 37, 36100 Vicenza, Italy; ^2^Department of Medicine DIMED, University of Padova Medical School, Via Giustiniani 2, 35100 Padova, Italy; ^3^Laboratory of Experimental Hepatology, Department of Medicine, University of Padova, Via Giustiniani 2, 35100 Padova, Italy; ^4^Department of Information Engineering, University of Padua, Via Gradenigo 6, 35131 Padova, Italy; ^5^Intensive Care Unit, San Bortolo Hospital, Via Rodolfi 37, 36100 Vicenza, Italy

## Abstract

*Background*. Cardiorenal Syndrome Type 5 (CRS Type 5) reflects concomitant cardiac and renal dysfunctions in the setting of a wide spectrum of systemic disorders. Our aim was to study *in vitro* effects of CRS Type 5 plasma on renal tubular cells (RTCs), in terms of cellular death and the characterization of inflammatory plasma profile in these patients. *Material and Methods*. We enrolled 11 CRS Type 5 patients from ICU and 16 healthy controls. Plasma from patients and controls was incubated with renal tubular cells (RTCs) and cell death was evaluated. Plasma cytokines were detected. *Results*. RTCs incubated with CRS Type 5 plasma showed significantly higher apoptosis and necrosis with respect to controls. Plasma cytokine profile of CRS Type 5 patients was significantly different from controls: we observed the production of pro- and anti-inflammatory mediators in these patients. Caspase-3, caspase-8, and caspase-9 were activated in cells treated with CRS Type 5 plasma compared to controls. *Conclusions*. Our results underline the cytotoxic effect of CRS Type 5 mediators on RTC viability, probably due to the activation of both intrinsic and extrinsic pathways of apoptosis and to the deregulation of cytokine release. The consequence may be the damage of distant organs which lead to the worsening of condition of patients.

## 1. Introduction

Heart and kidney are acting in tandem to regulate blood pressure, vascular tone, diuresis, natriuresis, intravascular volume homeostasis, peripheral tissue perfusion, and oxygenation through cellular and humoral signaling [1]. Unfortunately, combined heart and kidney dysfunction is very common [2]. This is the basis of the clinical condition defined as Cardiorenal Syndrome (CRS) [3].

The syndrome has been classified into five subtypes based on the organ primarily involved (heart or kidney) and on whether the failure is acute, chronic, or secondary [4].

Cardiorenal Syndrome Type 5 (CRS Type 5) reflects concomitant cardiac and renal dysfunctions in the setting of a wide spectrum of systemic disorders, like sepsis, infections, drugs, toxins, connective tissue disorders, diabetes mellitus, and systemic lupus erythematous.

In CRS Type 5 the heart and the kidney are both targets of a strong systemic inflammatory reaction [5] and there are marked cellular and molecular changes in these organs with a time-specific pattern. However, these aspects have not been sufficiently investigated. CRS Type 5 is characterized by generalized inflammatory response and by activation of coagulation and the fibrinolytic system and induces cellular and molecular changes in the heart and kidneys [6]. This inflammatory reaction is particularly relevant for sepsis, in which mechanisms of immune-mediated heart and kidney tissue injury have been described in detail [7]. In particular, several studies showed that inflammatory mediators, release of nitric oxide, and increased production of peroxynitrite are able to alter organ function and cause abnormal cell signaling, cell cycle arrest, and mitochondria dysfunction and can induce direct proapoptotic and proinflammatory effect on cardiomyocytes and kidney resident cells such as podocytes, endothelial cells, mesangial cells, and particularly tubular epithelial cells [5]. Furthermore, macrophages, neutrophils, and lymphocytes have been implicated in this syndrome [8]. Abnormalities in oxidative stress can be hypothesized to be the key mechanisms of CRS Type 5: high levels of oxygen radicals inactivate mitochondrial enzymes, cause DNA damage, and, by interacting with both DNA repair enzymes and transcription factors, lead to cell death [9].

The pathophysiology of CRS Type 5 is very complex and poorly understood as it involves several factors which are interrelated. Our aims were to examine* in vitro* that CRS Type 5 plasma was able to trigger a response in renal tubular cells (RTCs), resulting in apoptosis, and to characterize the inflammatory plasma profile of these patients.

## 2. Materials and Methods

### 2.1. Subjects

We enrolled 11 patients who developed CRS Type 5 in Intensive Care Unit (ICU). Demographic and clinical data of these patients were recorded and blood and urine biochemical parameter were analysed (Table 1).

CRS Type 5 was defined according to the current classification system [10]. Serum creatinine (SCr) was measured by Jaffè method and the eGFR was calculated with the 4-variable standardized-MDRD study equations [11]. AKI was defined by Acute Kidney Injury Network (AKIN) criteria [12]. None of the patients had exposure to contrast media in the 72 h preceding AKI. Sixteen healthy volunteers without systemic disorders or AKI or heart dysfunction were recruited as control group for this study.

### 2.2. Sample Collection

The procedures were in accordance with the Helsinki Declaration. All patients were informed about the experimental protocol and the objectives of the study before providing informed consent and blood sample. Peripheral venous blood samples were collected from all recruited patients within 4 hours of CRS Type 5 diagnosis into the ICU ward. The blood sample was collected in a EDTA-tube and subsequently centrifuged for 7 minutes at 3500 rpm. Plasma was immediately separated from the blood cells and stored at −80°C until use. All samples were processed within 2 hours after collection. Control samples from healthy volunteers were collected and processed in the same manners.

### 2.3. Determination of Plasma NGAL Concentration

The quantitative determination of plasma Neutrophil Gelatinase Associated Lipocalin (NGAL) of CRS Type 5 patients was performed by Alere Triage NGAL Test (Alere, San Diego, CA, USA), a point-of-care fluorescence immunoassay. Measuring the NGAL concentration was performed according to the manufacturer's protocol. NGAL concentrations were expressed as nanograms per milliliter (ng/mL). The measurable range of NGAL by this test is 15–1300 ng/mL. The analytical sensitivity of this assay is <15 ng/mL.

### 2.4. RTC Culture

Primary cultures of human proximal renal tubular epithelial cells were obtained from kidneys removed by surgical procedures from patients affected by renal carcinomas. An immortalized human proximal renal tubular epithelial cells (RTCs) line was generated by infection with a hybrid Adeno5/SV40 virus. The purity of primary cultures was assessed on the basis of cell characterization, according to published criteria [13]. RTCs were grown in complete liquid phase medium (RPMI 1640 with stable L-glutamine) supplemented with 10% heat-inactivated (30 min at 56°C) fetal bovine serum, 100 IU/mL penicillin, and 100 *μ*g/mL streptomycin (Sigma). RTCs were maintained in an incubator at controlled atmosphere (5% CO_2_) at 37°C and passaged at 80% confluence checked by inverted microscope.

### 2.5. Induction of Apoptosis

The RTCs were plated at 1.2 *∗* 10^4^ cells per well in 48-well plates and incubated with 90% RPMI 1640 medium (with 2 mM, L-glutamine, 100 IU/mL penicillin, and 100 mg/mL streptomycin) and 10% EDTA plasma from CRS Type 5 patients and healthy controls in standard condition (at 37°C in 5% CO2 for up to 24 h).

### 2.6. Evaluation of Apoptosis

#### 2.6.1. Annexin V and Propidium Iodide Detection Assay

Before incubation, all cells and cellular debris were collected and washed in Dulbecco's PBS (without calcium and magnesium) at pH 7.4. Cell viability, apoptosis, and necrosis were assessed using the Annexin V-FITC kit (Beckman Coulter, Brea, CA, USA) according to the manufacturer's protocol. This kit is based on the binding properties of annexin V to phosphatidylserine and on DNA-intercalating capabilities of propidium iodide (PI). Analysis was performed using a Navios flow cytometer (Beckman Coulter, Brea, CA, USA). Biparametric analysis revealed three distinct populations: viable cells with low FITC and low PI signals, apoptotic cells with high FITC and low PI signals, and necrotic cells with high FITC and high PI signals. We used negative controls (untreated RTCs): quadrants encompassed unstained cells, cells stained with annexin V-FITC alone (for FL-1 fluorescence), and cells stained with PI alone (detected in FL-4). A minimum of 15,000 events were collected for each sample.

### 2.7. Determination of Caspase-3, Caspase-8, and Caspase-9 Activity

RTCs were assayed for activation of caspase pathways. Caspase-3, caspase-8, and caspase-9 concentrations were measured by Human instant ELISA kits (eBioscience, San Diego, CA, USA) with a cytofluorometric assay. RTCs incubated for 24 h with plasma of CRS Type 5 patients and controls were processed according to the manufacturer's instructions and finally Caspase-3, caspase-8, and caspase-9 levels were measured in cell lysates by VICTOR X4 Multilabel Plate Reader (PerkinElmer Life Sciences, Waltham, MA, USA). The activity of caspases, assayed in duplicate wells, were calculated from the standard curve according to the manufacturer's protocol.

### 2.8. Cytokine Enzyme-Linked Immunosorbent Assay

The quantitative determination of IL-6, IL-1*β*, IL-8, IL-10, and IFN-*γ* in the plasma of CRS Type 5 patients and controls was performed by the Human Instant enzyme-linked immunosorbent assay (ELISA) kit (eBioscience, San Diego, CA). Cytokine determinations were performed according to the manufacturer's protocol and instructions. Optical density was read by VICTOR X4 Multilabel Plate Reader (PerkinElmer Life Sciences) at 450 nm. The amount of cytokines was calculated from the standard curve according to the manufacturer's protocol. All tests were performed in duplicate.

### 2.9. Statistical Analysis

Categorical variables were expressed as percentages; continuous variables were expressed as means ± standard deviation (parametric variables) or median and interquartile range (IQR) (nonparametric variables). The Mann-Whitney *U* test was used for comparison between two groups. Pearson's rank order correlation coefficient (rho) was used to test the correlation between variables. A *p* value < 0.05 was considered significant. Statistical analysis was performed using the SPSS (version 15; SPSS Inc., Chicago, IL, USA).

## 3. Results

### 3.1. CRS Type 5 Patients Characteristics

In Table 1 we collected demographic and laboratory data about patients enrolled for this study. Patients' clinical and biochemistry parameters were significantly different compared to controls.

The mean age of 11 patients with CRS Type 5 was 69.4 ± 10.8 years and 72.8% of these patients were male. The median baseline SCr of CRS Type 5 patients was 1.06 mg/dL (IQR 0.95–1.41); the median eGFR was 62 mL/min/1.73 m^2^ (IQR 50–75). Therefore, 20% of patients had an estimated glomerular filtration rate (eGFR) lower than 45 and 80% higher than 45 mL/min/1.73 m^2^.

No patients developed the need of mechanical ventilation but 4 patients needed continuous renal replacement therapy (CRRT) during this hospitalization period.

The mean age of 15 healthy volunteers was 52.0 ± 7.7 years and 47% of these subjects were male.

### 3.2. Plasma NGAL in CRS Type 5 Patients

Plasma NGAL was determined in CRS Type 5 patients at time of diagnosis and in healthy controls. NGAL levels were significantly higher in CRS Type 5 patients compared to control (*p* < 0.05). Specifically, the median value of NGAL in patients (612 ng/mL; IQR 158–1145) was around more than 10 times higher with respect to control subjects (60 ng/mL; IQR 60-60). We divided the study population into two groups: survivor (*n* = 8) and nonsurvivor (*n* = 3). NGAL value did not predict this outcome.

### 3.3. Effect of Plasma on RTC Viability

**Figure 1:**
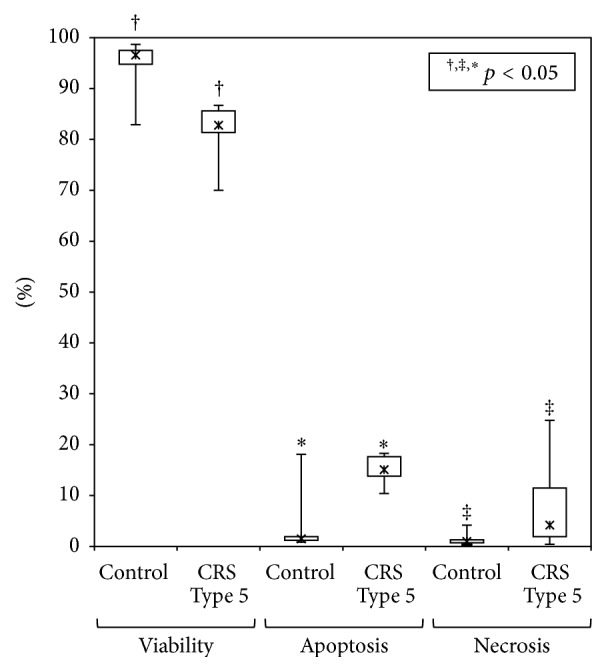
Evaluation of percentage of viability, necrosis, and apoptosis in RTCs after incubation with plasma from CRS Type 5 patients and healthy controls.

The RTCs incubated with plasma from CRS Type 5 patients showed significantly lower viability (82.8%; IQR 81.2–85.6) compared to cell incubated with plasma from healthy subjects (96.6%; IQR 94.8–97.5) (*p* < 0.05) (Figure 1).

Cells incubated with plasma from these patients showed higher apoptosis and necrosis rates compared with those incubated with controls plasma (*p* < 0.05). The level of apoptosis detected after 24 h incubation was 15.1% (IQR 13.8–17.6) in patients and 1.5% (IQR 1.2–1.9) in control group. Furthermore, the level of necrosis was 4.2% (IQR 2.25–11.5) in patients and 0.98% (IQR 0.7–1.3) in the control group (Figure 1).

### 3.4. Measurement of Caspases Activity

The activity of caspases was measured in RTCs treated for 24 h with plasma EDTA. In concordance with the apoptosis rate, RTCs incubated with CRS Type 5 plasma showed a significantly higher caspase-3 activity compared to those of controls (4.54, IQR 4.51–4.60 versus 1.04, IQR 0.92–1.57) (*p* < 0.01). These results were confirmed by a positive correlation between cell apoptosis and caspase-3 levels (rho = 0.71; *p* < 0.01). Moreover, a significantly higher activity of caspase-8 (0.96, IQR 0.91–1.04 versus 0.68, IQR 0.52–0.74) and caspase-9 (14.49, IQR 6.71–54.50 versus 4.17, IQR 2.28–5.48) was observed compared to controls (*p* < 0.01) (Table 2). Furthermore, caspase-3 levels showed a significantly positive correlation with caspase-8 (rho = 0.72, *p* < 0.05). A mild positive correlation was also observed between caspase-3 and caspase-9 (rho = 0.49, *p* < 0.05).

### 3.5. Evaluation of Plasma Immune-Mediated Molecules

To examine potential mediators involved in the immune-mediated damage in CRS Type 5 pathogenesis, plasma pro- and anti-inflammatory cytokine levels were measured by ELISA in patients and controls. IL-6, IL-10, IL-8, IL-1*β*, and IFN-*γ* levels were all significantly elevated in the CRS Type 5 group when compared with healthy control subjects (*p* < 0.05) (Table 3). Finally, statistical multivariate analysis showed a negative strong correlation between IL-1*β* levels in plasma and RTCs viability (rho = 0.91, *p* < 0.001). Furthermore, we divided CRS Type 5 patients into two group: patients who underwent CRRT (*n* = 4) and not (*n* = 7). There were no differences in the levels of immune-mediated molecules and in cell apoptosis, necrosis, and viability in the two groups (exception for IL-8 with *p* = 0.038) (Table 4).

## 4. Discussion

A growing body of research indicates that immune-mediated, inflammatory, and apoptotic mechanisms may play a role in the pathogenesis of CRS Type 5 [14].

In this study, we examined the role of inflammatory mediators and apoptotic mechanisms in the pathophysiology of CRS Type 5. We analysed the* in vitro* cytotoxic effects of CRS Type 5 plasma on RTCs when it was added to culture medium for 24 hours. In this way we reproduced in small scale what happened in renal tubules of CRS Type 5 patients when systemic disorders induced cardiac and renal dysfunctions.

We observed significantly increased* in vitro* levels of apoptosis and necrosis in RTCs incubated with plasma from CRS Type 5 patients compared to controls. Furthermore, we showed a marked proapoptotic activity trigged by both intrinsic and extrinsic pathways in RTCs incubated with plasma from CRS Type 5 patients. In concordance with the apoptosis rate, we observed a fourfold increase of caspase-3 activation in RTCs, which was associated with a higher extent of apoptotic death.

Plasma NGAL of CRS Type 5 patients was higher when compared to controls but there was not a statistic relationship between this parameter and outcome. Mortara et al. reported the relationship between the level of NGAL at admission and the kidney function, defined as baseline creatinine and eGFR [15]. These authors reported that NGAL measurements at admission did not predict worsening of renal function (WRF); on the contrary, several measurements of this marker in the first day of hospitalization can accurately predict WRF [15]. Some explanations of this discrepancy in NGAL role in different studies may lie in the utilization of different sample types for NGAL measurements (urine and plasma) with probably different pathophysiological mechanisms and different methods for NGAL detection with different sensibility and specificity. In any case, our results on NGAL values and its connection with outcome were limited for the small size of the study: in fact, a minimum of 25 events are required to draw reliable conclusion in case of outcome analysis. In particular, Ronco et al. [16] proposed that plasma NGAL may be useful in the diagnosis and prevention of CRS if a curve of plasma values rather than a single plasma measurement is determined.

Subsequently, RTCs were incubated* in vitro* with plasma of CRS Type 5 patients and showed higher apoptosis and necrosis and lower viability when compared to cells incubated with control plasma. A decrease of viability was observed also by Virzi et al. [17] in monocytes incubated with plasma of CRS Type 1 patients. Mariano et al. [18] observed proapoptotic effect in tubular cells incubated with plasma from septic patients. Furthermore, RTCs apoptosis and necrosis were observed in CRS Type 1 both in human and in animal kidney during AKI [19, 20]. Probably, cellular apoptosis occurs in all CRSs and involves different types of cells and, therefore, different tissues. Animal and human models underline that tubular epithelial cells are susceptible to apoptosis and injury may contribute to organ failure [19, 20].

Wan and coworkers in 2003 showed that the mechanisms of tissue injury related to systemic disorders, such as sepsis, seem to be related to a direct activity induced by circulating mediators with both pro- and anti-inflammatory properties able to interact in a dynamic manner and to induce multiple organ failure [21].

In this study we founded high concentration of pro- and anti-inflammatory cytokines in CRS Type 5 plasma. Based on this result, we might hypothesize that apoptosis and organ damage were induced by these inflammatory mediators. Cytokines were produced by different tissues and cleared by the kidney [22]. In CRS Type 5 contest, systemic disorder was often accompanied by systemic inflammation. A large amount of inflammatory mediators were circulating in the body. This preliminary study provided another evidence for the causal role of circulating inflammatory mediators. Particularly, we observed a strong imbalance and disequilibrium in production and activation of pro- and anti-inflammatory mediators. An alteration in the regulation of cytokines may negatively affect the mechanism of host defense and render the host vulnerable. In fact, the host response and the immune mechanisms require fine balances between pro- and anti-inflammatory components and between recruitment and death of immunocompetent cells, involving lymphocytes and monocytes. Based on these preliminary results, we may speculate that CRS Type 5 could result from an uncontrolled and deregulated inflammatory response.

In fact, Bongartz and colleagues outlined the four major cardiorenal connectors: increased activity of rennin-angiotensin system, oxidative stress, high activity of the sympathetic nervous system, and, in particular, inflammation and cytokines [23].

In addition, we observed a significant difference in IL-8 levels in patients who required CRRT and who did not. This finding suggests that patients who required CRRT might have a stronger inflammatory disequilibrium. However, the observed increase in levels of this cytokines should not necessarily be interpreted as a central role for IL-8 in CRRT. Therefore, other experiments and a higher sample size are necessary to better understand inflammatory aspects of CRS Type 5, CRRT, and the humoral signalling.

Moreover, we investigated the role of caspases, in particular caspase-3, caspase-8, and caspase-9, in apoptotic activation in CRS Type 5 to better understand the apoptotic pathway involved. Caspase-3 is activated by the upstream caspase-8 and caspase-9, since it serves as a convergence point for different signalling pathways. In particular, caspase-3 is an effector caspase able to cleave various cytoplasmic or nuclear substrates, which led to many morphological features of apoptotic cell death. Caspase-8 is an intracellular cysteine protease which plays a central role in the initiation of apoptotic cascades and caspase-9 is the most upstream member of the apoptotic protease cascade that is triggered by cytochrome c and ATP. We considered the detection of caspase-8 and caspase-9 representative of activation of extrinsic and intrinsic pathway, respectively. Our results showed that both caspases were activated in RTCs incubated with CRS Type 5 plasma. Therefore, we hypothesize a dual apoptotic signal activation: intrinsic and extrinsic pathway may be induced in CRS Type 5 mechanism.

Even though both apoptotic signals were activated, it was not possible to understand if the two different pathways were trigged independently or not. Grunnet et al. [24] showed that the extrinsic pathway may activate the intrinsic pathway to induce cell death through caspase-8-mediated cleavage of the BH3-only Bcl-2 protein Bid. Cellular death may be activated by proinflammatory cytokines, and apoptotic signals may be triggered by different types of receptors, amplified downstream and converged, probably, in activation of NFkB. The main transcriptor factor induced cell death and inflammatory cytokine expression [25].

The results of our study warrant further determinations and more detailed mechanistic investigation of inflammatory mechanisms in CRS Type 5 patients that may contribute to gradual toxic injury in renal cells. Future investigations of tubular damage markers and inflammatory cytokine levels in patients plasma are required to determine cellular and humoral factor expression that engenders immune-mediated imbalances. This study has some limitations which should be taken into account when interpreting the results. This study is limited by the restricted number of patients and by the* in vitro* experimental evaluations. This* in vitro* model was performed to simplify experimental variables and to isolate different components and study them in well-controlled and reproducible conditions. It can be very challenging to extrapolate from the results of* in vitro* model back to the biology of the intact organism as a whole. Furthermore, for our experiments, we used human renal tubular cell line immortalized by Adeno5/SV40 virus.* In vivo*, the response of these cells could be partially different because of the presence of multiple different cell types, cell-cell interactions, extracellular matrix interactions, and the physiological context.

Furthermore, we evaluated plasma concentration of NGAL and many cytokines and we speculated about activation of cell signals pathway and the regulation of gene expression: our preliminary results can be considered as hypothesis-generating and stimulate further exploration of novel pathophysiological mechanisms in CRS Type 5.

## 5. Conclusion

In this study we examined* in vitro* effects of CRS Type 5 plasma on RTCs and specific cytokine profile of these patients. We observed a strong cytokine dysregulation with the enhanced production of plasma proinflammatory factors in CRS Type 5 patients with the increases in apoptosis rate in RTCs incubated with this plasma.

Our study demonstrated the induction of cellular death in RTCs incubated with CRS Type 5 plasma. In particular, we showed the activation of two distinct apoptotic pathways which could be proved to be an important contribution of the development of CRS Type 5 and might play a role in the pathophysiological mechanism of this syndrome. Induced apoptosis and inflammation could be essential for the pathophysiological organ cross talk and the distant organ damage in CRS Type 5.

Gaining a clear mechanistic understanding on how dysfunction of heart can lead to kidney organ failure is critical to identify potential treatment targets and disease management strategies.

## Figures and Tables

**Table 1:** Clinical and biochemistry parameter about CRS Type 5 patients enrolled.

	CRS Type 5 patients
Female (*n*)	3/11
Age (years)	69.4 ± 10.8
Creatinine baseline (mg/dL)	1.06 (0.95–1.41)
eGFR baseline (mL/min/1.73 m^2^)	62 (50–75)
Creatinine admission (mg/dL)	1.85 (0.77–3.64)
eGFR admission (mL/min/1.73 m^2^)	36 (17.5–86)
Temperature (°C)	38.3 (36.1–38.9)
Urine output (mL/day)	3040 (1542–3225)
Urea (mg/dl)	91 (59–192)
Na (mEq/L)	138 (135–144)
K (mEq/L)	3.9 (3.67–4.1)
White blood cells (/mm^3^)	10.8 (7.25–14.2)
Platelets (/mm^3^)	133 (109–149)
PaO_2_/FiO_2_	241.5 (194.5–273.5)
Nonsurvivors (*n*)	3/11
CRRT (*n*)	4/11

**Table 2:** Evaluation of caspase-3, caspase-8, and caspase-9 in RTCs after incubation with CRS Type 5 plasma and controls.

	CRS Type 5	Controls	*p* value
Caspase-3 (ng/mL)	4.54, IQR (4.51–4.60)	1.04, IQR (0.92–1.57)	<0.01
Caspase-8 (ng/mL)	0.96, IQR (0.91–1.04)	0.68, IQR (0.52–0.74)	<0.01
Caspase-9 (ng/mL)	14.49, IQR (6.71–54.5)	4.17, IQR (2.28–5.48)	<0.01

**Table 3:** Evaluation of immune-mediated molecules in CRS Type 5 plasma and controls.

Parameters	CRS Type 5	Controls	*p* value
IL-6 (pg/mL)	64.6 (59.3–83.8)	5.9 (3.5–7.3)	<0.05
IL-10 (pg/mL)	19.6 (8.1–54.6)	3.1 (2.0–3.9)	<0.05
IL-8 (ng/mL)	87.1 (78.0–119.5)	20.3 (12.6–47.6)	<0.05
IL-1*β* (pg/mL)	80.8 (45.9–273.9)	8.3 (4.3–41.1)	<0.05
IFN-*γ* (pg/mL)	17.9 (16.9–20.8)	2.0 (1.4–3.2)	<0.05

**Table 4:** Differences in immune-mediated molecules and *in vitro* viability rates in patients who have undergone CRRT or who did not.

*n*	no CRRT	CRRT	*p* value
	*n* = 7	*n* = 4	
IL-6 (pg/mL)	59.6 (57.3–79.4)	128.8 (98.1–155.3)	0.059
IL-10 (pg/mL)	19.6 (9.8–54.6)	16.3 (7.9–86.6)	0.850
IL-8 (ng/mL)	78.1 (77.5–86.4)	119.5 (95.1–341.5)	0.038
IL-1*β* (pg/mL)	80.8 (45.1–233.3)	111.3 (49.8–525.6)	0.450
IFN-*γ* (pg/mL)	19.7 (17.5–30.1)	17.4 (16.9–17.8)	0.257
Viability (%)	82.8 (81.5–85.6)	83.1 (78.4–85.4)	0.775
Apoptosis (%)	15.1 (13.9–17.1)	15.8 (13.7–17.9)	0.850
Necrosis (%)	4.1 (2.2–11.5)	6.2 (3.2–12.3)	0.850
